# Asiatic Acid Attenuates Inflammation Induced by *Salmonella* via Upregulating LncRNA TVX1 in Microglia

**DOI:** 10.3390/ijms231810978

**Published:** 2022-09-19

**Authors:** Wenshu Zou, Jingyan Zhang, Kai Zhang, Zhiping Peng, Ruihua Xin, Lei Wang, Jianxi Li

**Affiliations:** Engineering and Technology Research Center of Traditional Chinese Veterinary Medicine of Gansu Province, Key Laboratory of Veterinary Pharmaceutical Development of Ministry of Agriculture and Rural Affairs of China, Lanzhou Institute of Husbandry and Pharmaceutical Sciences Chinese Academy of Agricultural Sciences, Lanzhou 730050, China

**Keywords:** *Salmonella typhimurium* infection, Asiatic acid, inflammation, LncRNA

## Abstract

*Salmonella typhimurium (S.T)* induces damage to the central nervous system; however, the role of Asiatic acid (AA) in this is still unknown. Microglia play a role as macrophages to recognize the invaded pathogenic microbes in the brain. The aim of this study was to investigate the protective effect and mechanism of AA on the central nervous system through an in vitro model of *S.T* infection in microglia. We pre-treated microglia with AA before *S.T* infection and explored the anti-infection mechanism of AA by sequencing, quantitative reverse transcription PCR (RT-qPCR), and Western blotting. Long non-coding RNA (lncRNA) sequencing demonstrated that inflammation is a major factor in *S.T* infection of microglia. RT-qPCR data demonstrated that AA inhibited *S.T*-induced increases in the mRNA levels of the pro-inflammatory factors *interleukin (IL)-1β*, *IL-6,* and *IL-18*. Western blotting demonstrated that AA inhibited *S.T*-induced activation of the nuclear factor (NF)-κB pathway and activation of the NLR family, pyrin domain-containing 3 (NLRP3) inflammasome. Expression of the lncRNA TVX1 in microglia was decreased by *S.T* infection and increased by pretreatment with AA. Inhibition of TVX1 expression reversed the anti-inflammatory effect of AA, and overexpression of TVX1 in microglia suppressed *S.T*-induced inflammation. In conclusion, AA attenuated *S.T*-induced microglial inflammation by upregulating the expression of the lncRNA TVX1.

## 1. Introduction

*Salmonella* poisoning is the most common bacterial food poisoning worldwide, and its prevalence increases yearly [1]. *Salmonella* pathogenicity island (SPI)1 and SPI2 encode two distinct type III secretion systems (T3SSs), and each of them a specific group of bacterial effector proteins into host cells [2]. SPI1-dependent translocation of bacterial invasion factors into host epithelial cells enables *Salmonella* to penetrate the small intestines [3]. The effector protein encoded by SPI2 promotes the survival of bacteria in *Salmonella*-containing vacuoles (SCVs) in macrophages, which can cause secondary systemic infection [4]. Early in *Salmonella* infection, cytoskeleton rearrangement can be induced, and then small GTP binding proteins on the cell surface interact with bacterial effectors to strictly regulate bacterial invasion [5]. SPI encoded effector proteins, such as guanine nucleotide exchange factors (GEFs), transform inactive GDP into active GTP [6], which regulates the nuclear factor (NF)-κB pathway, c-Jun N-terminal kinase (JNK), and p38-mitogen-activated protein, the NLR family, pyrin domain-containing 3 (NLRP3) inflammasome, and phagocytosis of the nicotinamide adenine dinucleotide phosphate (NADPH) oxidase complex [7,8,9]. Activation of various inflammation-related signaling pathways promotes the release of many cytokines; therefore, effective prevention of *Salmonella* infection is crucial.

Microglia are key players in brain development and homeostasis and play a role in maintaining the blood–brain barrier and protecting against brain diseases [10,11]. Microglia, as macrophages in the brain, can rapidly change from resting M0 to activated M1 or M2 cells when stimulated by brain injury, pathogen invasion, lipopolysaccharide, interferon (IFN), or tumor necrosis factor (TNF)-α [12,13,14]. Activation is characterized by cytoskeleton rearrangement, migration, enhancement of phagocytic ability, and release of a large number of cytokines [15]. In addition, abnormal activation of microglia is involved in the initiation and development of some central nervous system diseases, such as herpes simplex virus infection [16], Alzheimer’s disease [17], Parkinson’s disease, and autism spectrum disorder. Long non-coding RNAs (lncRNAs) have important roles in regulating microglia inflammation and responding to diseases of the nervous system. The lncRNA, HOTAIR, can prevent microglia from transforming to the pro-inflammatory M1-like phenotype [18]. The lncRNA, TUG1, regulates microglia polarization, production of inflammatory cytokines, and NF-κB transcription [19]. The lncRNA, 1810034E14Rik, reduces microglia activation in experimental ischemic stroke [20]. Therefore, a comprehensive understanding of the regulation of lncRNA RNAs in microglia may provide effective means for reducing inflammation. In this study, we systematically examined the global changes in lncRNA expression in microglia infected with *Salmonella typhimurium (S.T).* We aimed to explore the mechanism of *S.T* infection and discover effective preventions for *S.T* infection.

Asiatic acid (AA) is a pentacyclic triterpene that occurs naturally in the *Centella* species and has high dietary and medicinal value. AA can protect the nervous system; however, it remains unknown whether AA can protect microglia against *S.T* invasion. Zhang et al. showed that AA protects primary neurons from injury by antagonizing the mitochondrial pathway of apoptosis [21]. Krishnamurthy et al. found that AA reduced brain infarct volume and attenuated behavioral deficits in a mouse model of cerebral ischemia [22]. Lee et al. demonstrated that AA reduces nerve damage by altering mitochondrial function in rats [23]. Therefore, the aim of this study was to investigate the global lncRNA expression changes in *S.T*-infected microglia in response to AA treatment. Our data provided some evidences for developing new therapeutics for *S.T* infection.

## 2. Results

### 2.1. Overview of LncRNA Sequencing

All raw data can be found in NCBI (Bio Project ID: PRJNA765141). A total of 700,566,875 raw reads (260,063,128 for control, 262,848,792 for *S.T* group, and 177,654,955 for AA + *S.T* group) were generated. The low-quality, linker, and unknown base reads were filtered out to leave 744,531,748 clean reads (252,542,660 for control, 239,464,494 for *S.T* group, and 252,524,594 for AA + *S.T* group) (Figure S1). The error rate of each base in lncRNA-seq was less than 5% (Figure S2). GC content varies among species, but the 6 bp random primer used in the reverse transcription process causes a certain deviation in the composition of the first few nucleotides, which tend to stabilize after normal fluctuations (Figure S3). Other standards, Q20% and Q30%, also met standard criteria (Table S2).

### 2.2. Differential Analysis of LncRNA Expression

**Figure 1:**
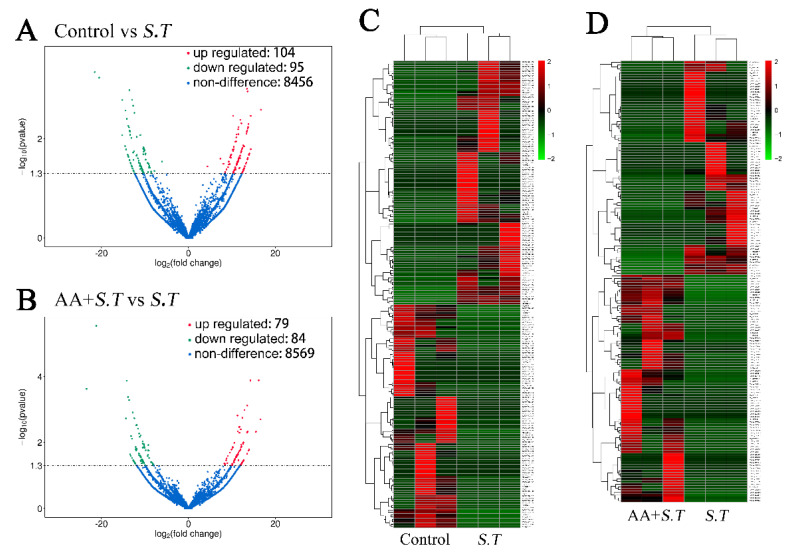
Differentially Expressed Transcripts and Gene Profiles. Volcano plots showing lncRNAs in microglia. (**A**) Significantly differentially expressed lncRNAs between *S.T* and control. (**B**) Significantly differentially expressed lncRNAs between *S.T* group and AA + *S.T* group (*n* = 3, *p* < 0.05). Heatmaps showing significant differential lncRNA expression in microglia. (**C**) Differentially expressed lncRNAs between *S.T* group and control. (**D**) Differentially expressed lncRNAs between *S.T* group and AA + *S.T* group. (*n* = 3, *p* < 0.05). Data represent the control, *S.T* group and AA + *S.T* group, *n* = 3 per group.

The expression levels of lncRNA transcripts were assessed using FPKM values. There were 199 significantly differentially expressed lncRNA transcripts between *S.T* group and control, 104 transcripts were upregulated and 95 were downregulated (*p* < 0.05). There were 163 significantly differentially expressed lncRNAs between *S.T* group and AA + *S.T* group, with 79 upregulated transcripts and 84 downregulated transcripts (*p* < 0.05). A volcano map shows the different lncRNAs (Figure 1A,B). Cluster analysis of lncRNA expression was conducted, and a heatmap was constructed to visualize the results (Figure 1C,D).

To confirm the differential expression identified by RNA sequencing, we used real-time PCR and analyzed 12 lncRNAs and exhibited statistically significant differential expression (Figure S4). In summary, the quantitative reverse transcription PCR (RT-qPCR) results were highly consistent with the RNA sequencing data.

### 2.3. Functional Annotation: Gene Ontology (GO)

**Figure 2:**
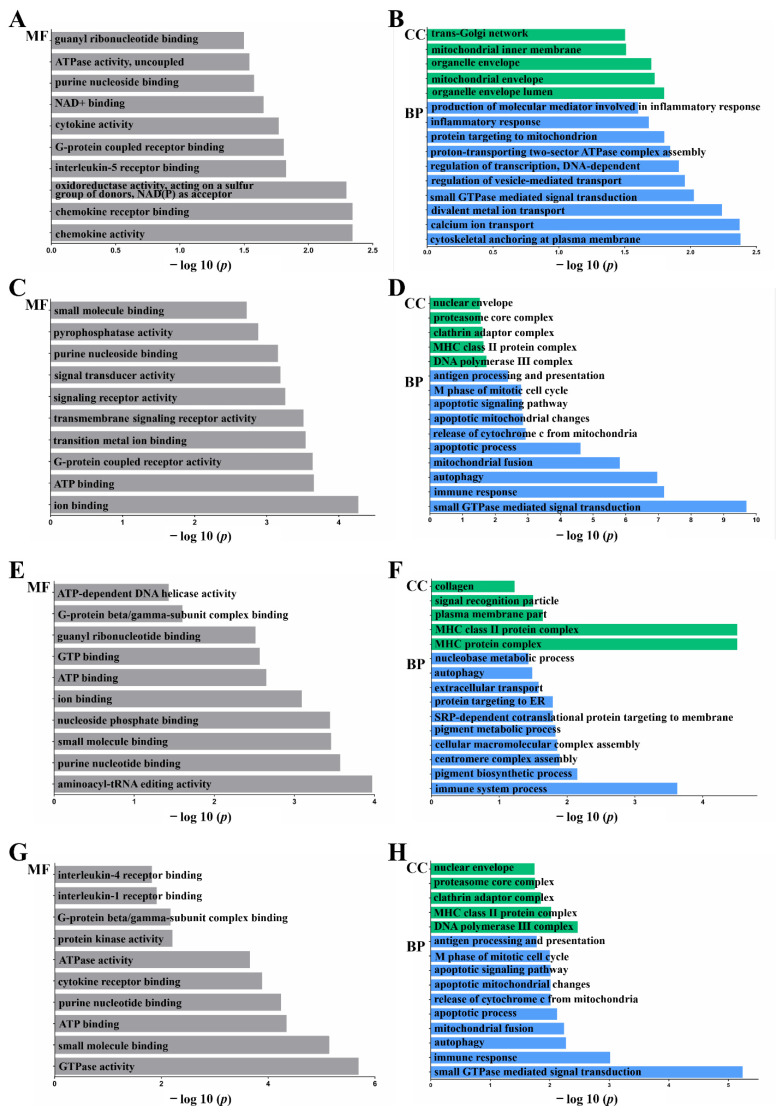
GO Terms of LncRNA Target mRNAs. GO annotation of biological process (BP), cellular component (CC), and molecular function (MF) terms of differentially expressed genes. Co-location prediction of lncRNAs between (**A**,**B**) *S.T* group and control, (**C**,**D**) *S.T* group and AA + *S.T* group. GO analysis was performed on mRNAs identified by searching for genes within 100 kb upstream and downstream of the lncRNA (*n* = 3, *p* < 0.05). Prediction of co-expression of lncRNAs between (**E**,**F**) *S.T* group and control, (**G**,**H**) *S.T* group and AA + *S.T* group. GO analysis was performed on mRNAs identified by analyzing expression correlations among multiple samples (*n* = 3, *p* < 0.05).

The enriched GO terms, biological process (BP), cellular component (CC), and molecular function (MF) are shown in Figure 2 (*p* < 0.05). In CC, genes were mainly enriched in the cytoskeleton (organelle membrane, integral to membrane, intrinsic to membrane, membrane part), Golgi (trans-Golgi network), mitochondrial (mitochondrial intermembrane space, mitochondrial envelope), and MHC protein-related terms (MHC class II protein complex, MHC protein complex) (*p* < 0.05).

In MF and BP, genes were mainly enriched in the immune process (autophagy, chemokine activity, cytokine receptor binding, interleukin-5 receptor binding, inflammatory response, interleukin 1 receptor binding, interleukin 4 receptor binding, apoptotic mitochondrial changes, apoptotic process, response to DNA damage stimulus, cellular response to stress, cellular response to stimulus, signaling receptor activity, immune response), GTPase (GTPase activity, small GTPase-mediated signal transduction, G-protein beta/gamma-subunit complex binding, G-protein coupled receptor binding), and cell communication-related terms (ATP binding, ATPase activity, ATP-dependent DNA helicase activity, ion binding, cell communication) (*p* < 0.05).

### 2.4. Functional Annotation: Kyoto Encyclopedia of Genes and Genomes (KEGG)

**Figure 3:**
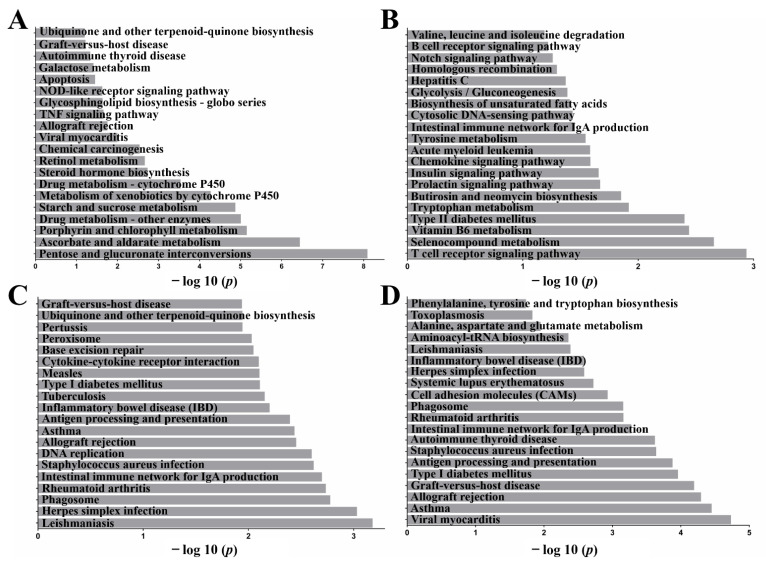
Pathways of the Top 20. The enrichment factor represents the ratio between the differentially expressed gene and all annotated lncRNA target genes enriched in the pathways of the top 20. Co-location prediction of lncRNAs between (**A**) *S.T* group and control, (**B**) *S.T* group and AA + *S.T* group. KEGG analysis was performed on the mRNAs identified by searching for genes within 100 kb upstream and downstream of the lncRNA. Prediction of co-expression of lncRNAs between (**C**) *S.T* group and control, (**D**) *S.T* group and AA + *S.T* group. KEGG analysis was performed on mRNAs obtained by analyzing expression correlations among multiple samples.

KEGG pathway analysis was conducted to determine the signaling cascades and pathways related to the top 20 (Figure 3). The immune system categories identified were autoimmune thyroid disease, apoptosis, allograft rejection, phagosome, rheumatoid arthritis, antigen processing and presentation, inflammatory bowel disease (IBD), intestinal immune network for IgA production, and T/B cell receptor signaling pathway. Common inflammatory pathways identified included TNF, NOD-like receptor, apoptotic, chemokine, Notch, and cytokine–cytokine receptor interaction (*p* < 0.05).

### 2.5. AA Decreased the Inflammation Induced by S.T by Upregulating TVX1

**Figure 4:**
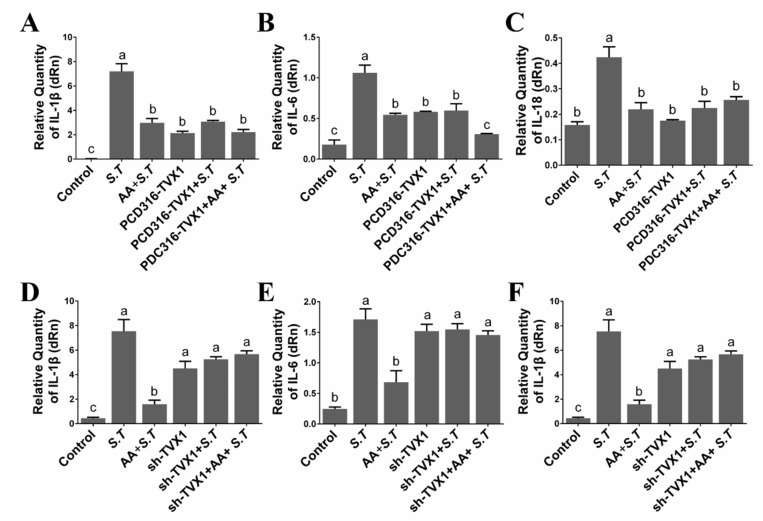
Effect of LncRNA TVX1 on AA Activation of Inflammatory Factors in *S.T*-infected Microglia. (**A**–**C**) Effect of lncRNA TVX1 overexpression plasmid PCD316-TVX1 on expression of *S.T*-induced pro-inflammatory factors (*IL-1β*, *IL-6, and IL-18*) in microglia (Different letters represent significant differences, *p* < 0.05, *n* = 3). (**D**–**F**) Effect of lncRNA TVX1 inhibitory plasmid sh-TVX1 on expression of *S.T*-induced pro-inflammatory factors *(IL-1β*, *IL-6, and IL-18)* in microglia (Different letters represent significant differences, *p* < 0.05, *n* = 3).

Both *S.T* infection and AA pretreatment affected the inflammatory response, as observed from GO and KEGG analysis. We, therefore, examined the expression of inflammation-related cytokines. RT-qPCR showed that the expression of pro-inflammatory factors, *IL-1β*, *IL-6,* and *IL-18* was decreased after AA pretreatment compared with *S.T* group infected microglia (Figure 4A–C), suggesting that AA inhibited the inflammation caused by *S.T*. Sequencing demonstrated a significant decrease in lncRNA TVX1 expression upon *S.T* infection compared with control, while a significant increase in response to AA pretreatment compared with *S.T* infection, and we found that lncRNA overexpression TVX1 plasmid PCD316-TVX1 inhibited *S.T* infection, decreased the expression of pro-inflammatory factors compared with *S.T* group (Figure 4A–C), demonstrating that AA regulates *S.T* infection by upregulating lncRNA.

While lncRNA TVX1 inhibitory plasmid shRNA-TVX1 reversed the anti-inflammatory effect of AA, it increased the expression of pro-inflammatory factors compared with AA + *S.T* group (Figure 4D–F), demonstrating that AA inhibits *S.T*-induced microglial inflammation by promoting the transcription of lncRNA TVX1.

### 2.6. AA Inactivates the S.T-Induced NF-κB Pathway by Upregulating TVX1

**Figure 5:**
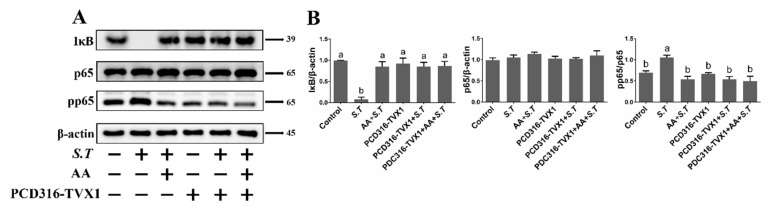
Effect of PCD316-TVX1 on AA Activation of the NF-κB Pathway in *S.T*-infected Microglia. Western blotting detection of NF-κB pathway protein changes in microglia. (**A**) Microglia were transfected with PCD316-TVX1 plasmid. Twenty-four hours later, cells were pre-treated with AA for one hour, then infected with *S.T*, and proteins were extracted for analysis. (**B**) NF-κB pathway protein grayscale images (Different letters represent significant differences, *p* < 0.05, *n* = 3).

To further explore the mechanism of AA-related regulation of inflammation, key proteins in the NF-κB signaling cascade were examined. NF-κB pathway activation is caused by the degradation of IκB by E3 ubiquitin ligase, thereby inducing phosphorylation of nuclear p65. Western blotting showed that IκB levels decreased and pp65 levels increased after *S.T* infection relative to the control group (Figure 5), while IκB levels increased and pp65 levels decreased after pretreatment with AA relative to *S.T*-infected cells (Figure 5), demonstrating that AA interfered with *S.T*-induced NF-κB pathway activation.

**Figure 6:**
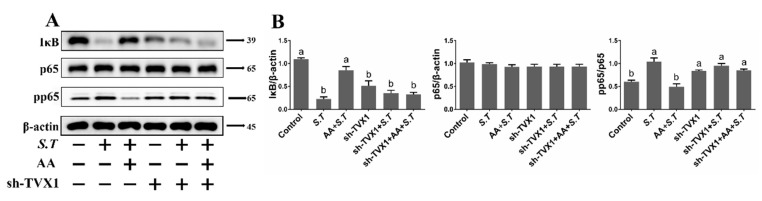
Effect of sh-TVX1 on AA Activation of the NF-κB Pathway in *S.T*-infected Microglia. Western blotting detection of NF-κB pathway protein changes in microglia. (**A**) Microglia were transfected with sh-TVX1 plasmid. Twenty-four hours later, cells were pre-treated with AA for one hour, then infected with *S.T*, and proteins extracted for analysis. (**B**) NF-κB pathway protein grayscale images (Different letters represent significant differences, *p* < 0.05, *n* = 3).

To demonstrate the regulatory role of lncRNA TVX1 on the NF-κB pathway in *S.T* infected and AA-treated cells, we transfected plasmids with lncRNA TVX1 overexpression plasmid PCD316-TVX1 and lncRNA TVX1 suppressor plasmid sh-TVX1. IκB levels were elevated and pp65 levels were decreased after PCD316-TVX1 transfection compared with *S.T*-infected cells, demonstrating that PCD316-TVX1 inhibited *S.T*-induced activation of the NF-κB pathway (Figure 5). In contrast, sh-TVX1 transfection decreased IκB levels and increased pp65 levels compared with AA + *S.T* group (Figure 6), demonstrating that *S.T* infection promotes inflammation by downregulating TVX1 to activate the NF-κB pathway, while AA inhibits the NF-κB pathway by upregulating TVX1 to reduce the inflammatory response.

### 2.7. AA Inactivates the S.T-Induced NLRP3 Inflammasome by Upregulating TVX1

**Figure 7:**
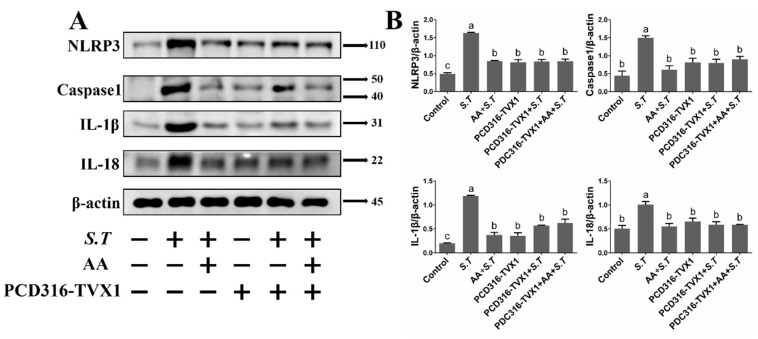
Effect of PCD316-TVX1 on AA Activation of the NLRP3 Inflammasome in *S.T*-Infected Microglia. Western blotting detection of NLRP3 inflammasome protein changes in microglia. (**A**) Microglia were transfected with PCD316-TVX1 plasmid. Twenty-four hours later, cells were pre-treated with AA for one hour, then infected with *S.T*, and proteins extracted for analysis. (**B**) NLRP3 inflammasome protein grayscale images (Different letters represent significant differences, *p* < 0.05, *n* = 3).

Activation of the NF-κB pathway promotes increased NLRP3 expression, which allows NLRP3 to recruit intracellular caspase 1. This promotes cleavage and maturation of NLRP3, which in turn induces the release of *IL-1β* and *IL-18* to exacerbate the inflammatory response. Our previous experiments have demonstrated that *IL-1β* and *IL-18* mRNA levels increase after *S.T* infection, which was prevented by AA; however, the mechanism of NLRP3 regulation is not clear. Detection of NLRP3 pathway proteins revealed increased levels of NLRP3 and caspase 1, along with increased levels of *IL-1β* and *IL-18* after *S.T* infection compared with controls, demonstrating that *S.T* infection activated the NLRP3 inflammasome in microglia (Figure 7). AA inhibited the expression of NLRP3, caspase 1, *IL-1β* and *IL-18* (Figure 7), indicating that AA reduced *S.T*-induced microglia inflammation by inhibiting activation of the NLRP3 inflammasome.

**Figure 8:**
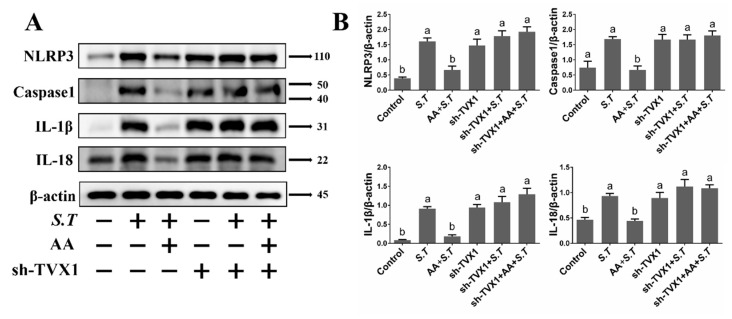
Effect of sh-TVX1 on AA Activation of the NLRP3 Inflammasome in *S.T*-infected Microglia. Western blotting detection of NLRP3 inflammasome protein changes in microglia. (**A**) Microglia were transfected with sh-TVX1 plasmid. Twenty-four hours later, cells were pre-treated with AA for one hour, then infected with *S.T*, and proteins extracted for analysis. (**B**) NLRP3 inflammasome protein grayscale images (Different letters represent significant differences, *p* < 0.05, *n* = 3).

Transfection of the overexpression plasmid PCD316-TVX1 inhibited *S.T*-induced activation of the NLRP3 inflammasome compared with AA + *S.T* group (Figure 7), similar to the effect of AA pretreatment. Transfection of the suppression plasmid sh-TVX1 activated the NLRP3 inflammasome compared with AA + *S.T* group (Figure 8), demonstrating that *S.T* promotes inflammation by downregulating TVX1 to activate the NLRP3 inflammasome, while AA reduces the inflammatory response by upregulating TVX1 to inhibit the NLRP3 inflammasome.

## 3. Discussion

The mechanism of central nervous system inflammation caused by *Salmonella* is still a mystery. Microglia cells in the brain act as macrophages to recognize *S.T* infection. GO and KEGG analysis of microglia sequencing data revealed inflammation and immunity to be the main consequences of *S.T* infection. The activated cell surface receptor, toll-like receptor (TLR), recruits intracellular MyD88, which is followed by phosphorylation of IκB and nuclear translocation of p65, leading to increased *IL-1β* transcription [24]. Cytokines of the IL-1 family are key mediators of peripheral and central inflammatory responses. Pro-*IL-1β* is mainly produced by resident macrophages and requires cleavage by caspase 1 to become active *IL-1β* [25]. Excessive *IL-1β* promotes activation of the NF-κB pathway and induces the transcription and release of other cytokines, such as IL-6 and TNF-α [26]. Moreover, pro-*IL-18* also needs caspase 1 cleavage to produce its mature form. *IL-18* combined with IFNγ and *IL-12* promotes Th1 responsiveness [27]. All this evidence suggests that the role of factors of the IL family in inflammation cannot be ignored. Here, the expression of *IL-1β*, *IL-6, and IL-18* mRNA was significantly increased in microglia after *S.T* infection, demonstrating that *S.T* infection promotes inflammation in microglia. AA significantly downregulated the expression of *IL-1β*, *IL-6, and IL-18*, demonstrating that AA reduced *S.T*-induced microglial inflammation.

The NLRP3 inflammasome is closely related to inflammation. NLRP3 inflammasome activation in response to *S.T* infection recruits caspase 1 to cleave *IL-1β* and *IL-18* to release inflammatory cytokines [28]. Direct inhibition of the NLRP3 inflammasome as an alternative approach to targeting *IL-1β* reduces the inflammatory response in gout [29]. Inhibition of the NLRP3 inflammasome reduces obesity-induced macrophage activation and thereby reduces inflammation [30]. Natural plant monomers, such as baicalin, curcumin, and andrographolide, alleviate inflammation by inhibiting the expression of the NLRP3 inflammasome [31,32,33]. Our study also demonstrated that AA inhibited *S.T*-induced elevated expression of NLRP3 and caspase 1. Further evidence showed that the expression of *IL-1β* and *IL-18*, a downstream protein of NLRP3 inflammasome, was reduced after AA pretreatment, which also demonstrated the anti-inflammatory effect of AA. A hallmark of NF-κB signaling pathway activation is the nuclear translocation of p65, which promotes the transcription of NLRP3 for inflammasome activation [34]. In the present study, AA pretreatment reduced *S.T*-induced activation of the NF-κB pathway and the NLRP3 inflammasome, further demonstrating the anti-inflammatory effect of AA.

Our sequencing data showed that lncRNA TVX1 expression was decreased upon *S.T* infection and increased upon AA pretreatment. Many reports have demonstrated that regulating the expression of lnc RNAs exerts anti-inflammatory effects. Non-coding RNAs alleviate neuroinflammation by downregulating the TLR4/NF-κB pathway by inhibiting activation of the NLRP3 inflammasome [35]. In the present study, we found that overexpression of lncRNA TVX1 significantly inhibited *S.T*-induced changes in cytokines and activation of the NF-κB pathway and NLRP3 inflammasome. Inhibition of lncRNA TVX1 expression reversed the anti-inflammatory effect of AA with increased expression of pro-inflammatory factors, and activation of the NF-κB pathway and NLRP3 inflammasome.

## 4. Materials and Methods

### 4.1. Cell Culture

The immortalized murine microglia cell line, BV-2, was purchased from Procell Life Science & Technology Co., Ltd. (CL-0493, Wuhan, China). Cells were cultured in Dulbecco’s Modified Eagle’s Medium (DMEM) supplemented with 10% fetal bovine serum (Gibco, Grand Island, NY, USA) and grown in a 37 °C humidified incubator under 95% air and 5% CO_2_.

### 4.2. Infection and AA Treatment of Microglia

Cells were seeded onto tissue culture plates and infected with *Salmonella typhimurium (S.T)* SL1344 (ATCC14028, National Center for Medical Culture Collections, Beijing, China). Bacterial culture was added to cells at a multiplicity of infection (MOI) of 100. One hour later, extracellular bacteria were removed and cells were incubated for one hour in medium containing 100 μg/mL gentamicin. Cells were then washed and subsequently cultured in medium containing less gentamicin (20 μg/mL) for 22 h, then collected for subsequent experiments. A total of 12.5 μM of AA (>98% high-performance liquid chromatography (HPLC) purity) (Sigma-Aldrich, St. Louis, MO, USA) was added to microglia one hour before infection and removed during infection.

### 4.3. RNA Extraction, cDNA Library Preparation and Sequencing

Sequencing was performed by Tianjin Novogene Co., Ltd. (Tianjin, China). Total RNA of microglia was isolated using TRIzol (Invitrogen Life Technologies, Carlsbad, CA, USA), and RNA concentrations and quality were examined using a NanoDrop spectrophotometer (NanoDrop Technologies, Wilmington, DE, USA). The degradation and contamination of total RNA were assessed on 1% agarose gels.

cDNA libraries were prepared for lncRNA sequencing. Ribosomal RNA was removed from total RNA, and then the RNA was broken into 250–300 bp fragments. cDNA was synthesized using 6 bp random primers and the fragmented RNA as a template. The double-stranded cDNA was then end-repaired, and a tail was added and connected to the sequencing connector. The 350–400 bp cDNA was purified using AMPure XP beads (Beckman Coulter, Beverly, MA, USA). The cDNA library was generated by PCR amplification. The insert size of the library was determined using an Agilent 2100 Bioanalyzer (250–300 nt).

After sequencing, paired-end reads were generated. The FASTQ format raw reads were processed by in-house Perl scripts, and clean reads were obtained after removing low-quality reads. Q20, Q30, GC-content, and sequence duplication levels of the clean data were then calculated. The final clean reads were mapped to the mouse reference genome (https://ftp.ncbi.nlm.nih.gov/genomes/all/GCF/000/001/635/GCF_000001635.26_GRCm38.p6/GCF_000001635.26_GRCm38.p6_genomic.fna.gz) in 23 September 2020.

### 4.4. Transcriptome Assembly and lncRNA Identification

Genome reads were spliced into transcripts and quantified by stringtiev 1.3.3. Then, the transcripts of each sample were combined using cuffmerge v2.2.1, and transcripts with uncertain chain direction and transcripts of less than 200 nt were removed. lncRNAs were predicted by comparing the transcripts using cuffcompare v2.2.1. Coding potential was predicted using CNCI, CPC2 v3.2.0, and pfam_scan v1.3. lncRNAs and mRNAs were then compared; cuffcompare v2.2.1 was used to compare length and number, and getorf emboss-6.6 was used to compare open reading frames.

### 4.5. Analysis of Differential Expression

The fragments per kilobase of exon per million mapped fragments (FPKM) value is an index of the expression level of lncRNAs and protein-coding mRNAs. DEGseq R-2.15.3 was used to analyze the significance of expression difference, and *p* < 0.05 was considered significant. *p* < 0.05 was also used for drawing volcano and heat maps. Gene Ontology (GO) enrichment analysis was performed with goseq/topgo release 2.12. Kyoto encyclopedia of genes and genomes (KEGG) enrichment analysis was performed with kobas v2.0. Enrichment analysis included all predicted target genes of differentially expressed lncRNAs.

### 4.6. Plasmid Construction and Transfection

The lncRNA TVX1 (NCBI: XR_001785420.1, Mus musculus predicted gene, 32838 (Gm32838), transcript variant X1) overexpression and suppression plasmids were synthesized by XIEBHO BIO (Beijing, China). After amplification of the lncRNA TVX1 overexpression plasmid, NotI and HindIII restriction sites were introduced at either end, and the fragment was then combined with the plasmid pDC316, namely the lncRNA overexpression plasmid pDC316-TVX1. The lncRNA TVX1 inhibitory plasmid shRNA-TVX1 was double digested with AgeI and BamHI to recover a large fragment, namely the lncRNA suppressor plasmid sh-TVX1. Lipofectamine 2000 (Thermo Scientific, Waltham, MA, USA) was used for plasmid transfection following the manufacturer’s instructions. Cells were used for experiments 24 h after transfection. AA and *S.T* treatments are the same as previously described.

### 4.7. RT-qPCR

Complementary DNA was synthesized using a reverse transcription (RT) reagent kit with gDNA Eraser (Takara, Dalian, China). qPCR analysis was performed using TB Green^®^ Premix EX Taq™ II (Takara, Dalian, China) in accordance with the manufacturer’s instructions. β-actin was used as a normalization control. Quantification was performed using the comparative CT method (1000/2ΔCT, ΔCT = CT _target gene_ − CT _β-actin_). The gene-specific oligonucleotide primers used for qPCR are listed in Table S1.

### 4.8. Western Blotting Analysis

Total cellular proteins were separated by sodium dodecyl sulfate-polyacrylamide gel electrophoresis and then transferred to nitrocellulose membranes (PALL, New York, NY, USA). Membranes were then incubated with anti-IκB (#76041, Cell Signaling Technology, Danvers, MA, USA), anti-p65 (#8242, Cell Signaling Technology, Danvers, MA, USA), anti-phospho-p65 (#3033, Cell Signaling Technology, Danvers, MA, USA), anti-NLRP3 (#15101, Cell Signaling Technology, Danvers, MA, USA), anti-caspase 1 (#24232, Cell Signaling Technology, Danvers, MA, USA), anti-*IL-18* (#54943, Cell Signaling Technology, Danvers, MA, USA), anti-*IL-1β* (#12703, Cell Signaling Technology, Danvers, MA, USA), or anti-β-actin (66009-1, Proteintech, Wuhan, China) primary antibodies overnight at 4 °C. Goat anti-Mouse IgG (H + L)-HRP (PR30012, Proteintech, Wuhan, China) and Goat anti-Rabbit IgG (H + L)-HRP (PR30011, Proteintech, Wuhan, China) secondary antibodies were incubated for 1 h at room temperature. Target proteins were visualized with enhanced chemiluminescence (ECL) reagents (Advansta, San Jose, CA, USA).

### 4.9. Statistical Analysis

Statistical analysis was performed using SPSS software (version 20.0). Real-time PCR data are expressed as the mean ± standard error of the mean (SEM). One-way analysis of variance (ANOVA) and Tukey’s post hoc analysis were used for multigroup comparisons of the means. Densitometric values of immunoblot signals were obtained from three separate experiments using Image J software (National Institutes of Health, Bethesda, MD, USA).

## 5. Conclusions

Inflammation is the main consequence of *S.T* infection of microglia, manifested as increased expression of pro-inflammatory factors *IL-1β*, *IL-6, and IL-18* and activation of the NF-κB pathway and NLRP3 inflammasome. We found that AA pretreatment effectively reversed *S.T*-induced inflammation and reduced pro-inflammatory factors *IL-1β*, *IL-6, and IL-18* expression and the NF-κB pathway and NLRP3 inflammasome activation. The response after inhibition of lncRNA TVX1 expression in microglia is similar to S.T-induced inflammatory responses. AA pretreatment increased the expression of lncRNA TVX1, thereby reducing *S.T*-induced microglial inflammation.

## Data Availability

The sequencing data presented in this study are openly available in NCBI, reference number [Bio Project ID: PRJNA765141]. All the data used to support the findings of this study are presented in this article.

## Supplementary Materials

The following supporting information can be downloaded at: https://www.mdpi.com/article/10.3390/ijms231810978/s1.
