# Supplementary material for: Asiatic Acid Attenuates Inflammation Induced by Salmonella via Upregulating LncRNA TVX1 in Microglia
Source: Int J Mol Sci. 2022 Sep 19;23(18):10978. doi: 10.3390/ijms231810978 (PMC9504590; doi:10.3390/ijms231810978)
Supplement: Supplementary file 1 [file ijms-23-10978-s001.zip › ijms-1912233-Supplementary materials.pdf]

Asiatic Acid Attenuates Inflammation Induced by *Salmonella* via Upregulating LncRNA TVX1 in Microglia

Wenshu Zou, Jingyan Zhang, Kai Zhang, Zhiping Peng, Ruihua Xin, Lei Wang and Jianxi Li *

Engineering and Technology Research Center of Traditional Chinese Veterinary Medicine of Gansu Province, Key Laboratory of Veterinary Pharmaceutical Development of Ministry of Agriculture and Rural Affairs of China, Lanzhou Institute of Husbandry and Pharmaceutical Sciences Chinese Academy of Agricultural Sciences, Lanzhou 730050, China;

* Correspondence: lijianxi@caas.cn; Tel.: +86-09312115263

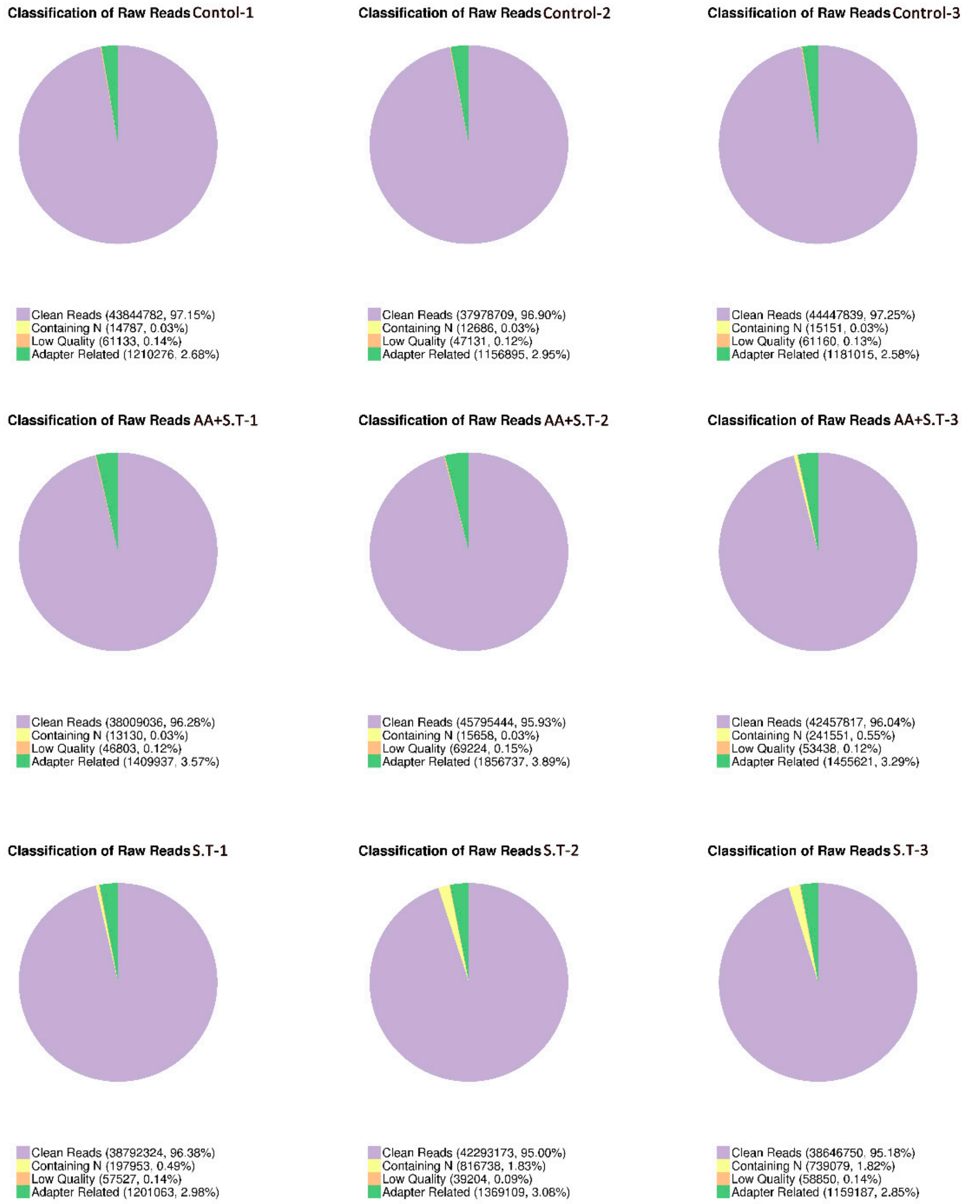

Figure S1. Raw data of lncRNA sequencing. Data represent the control, *S.T* and AA + *S.T*, n=3 per group.

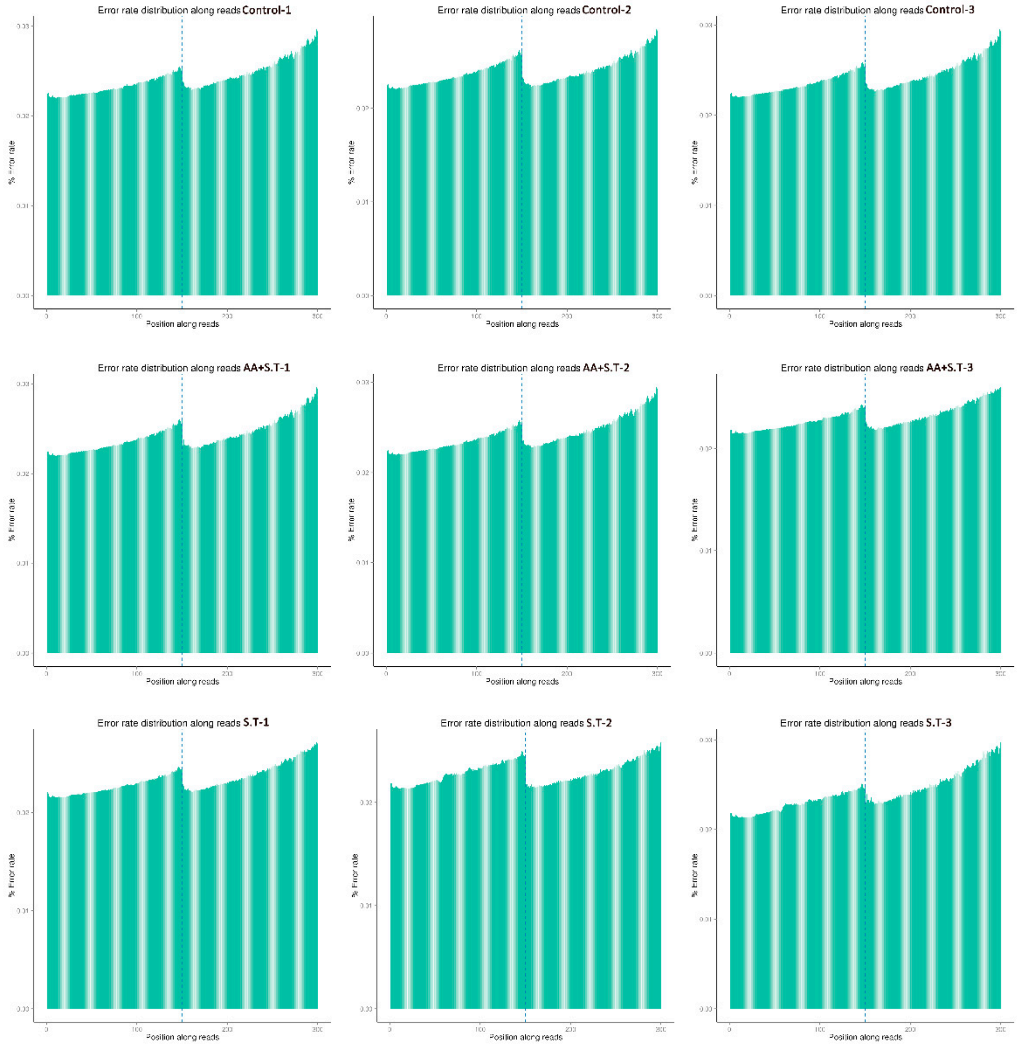

Figure S2. Error rate distribution map of lncRNA sequencing. X-axis is the base position of reads, Y-axis is the single base error rate. Data represent the control, *S.T* and AA + *S.T*, n=3 per group.

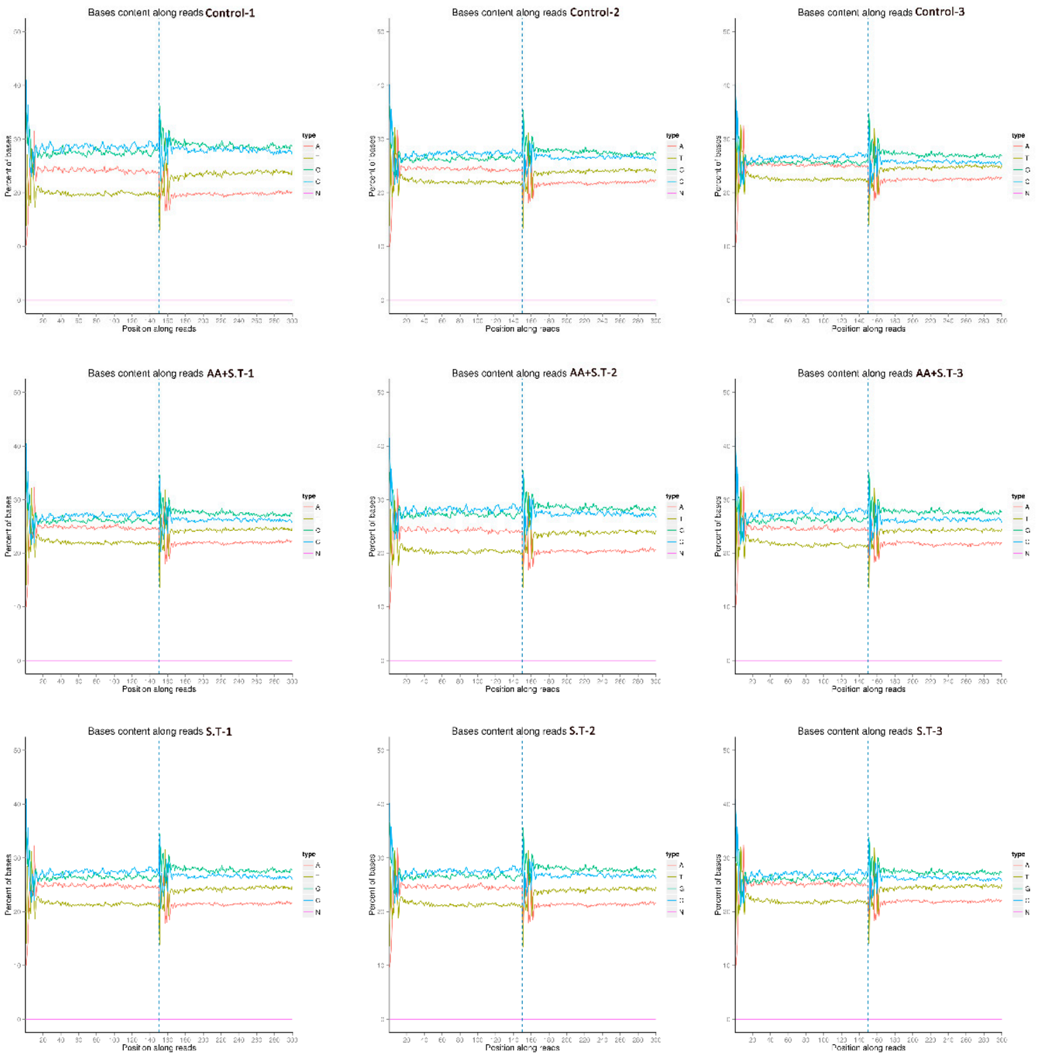

Figure S3. GC content of lncRNA sequencing. X-axis is the base position of reads, Y-axis is the proportion of the base. Data represent the control, *S.T* and AA + *S.T*, n=3 per group.

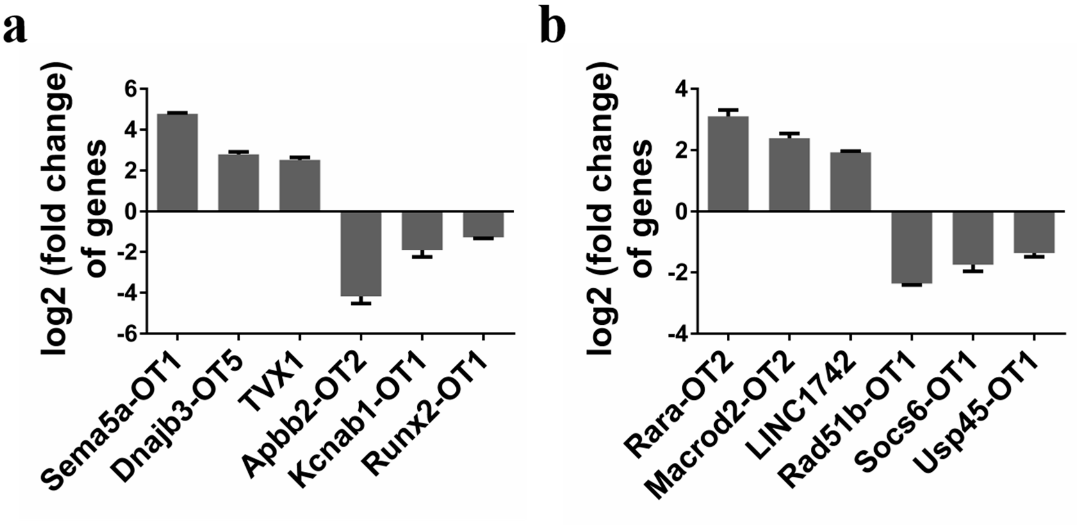

Figure S4 Verification of lncRNA sequencing results using qPCR.

(a) Differential lncRNAs between *S.T* and control. (b) Differential lncRNAs between *S.T* and AA + *S.T*.

Values are expressed as the mean ± SEM (n = 3 per group).

Table S1. The primers for RT-q PCR used in present study

Name	Primer sequence (5′ - 3′)
β-actin	F: CCACCATGTACCCAGGCATT<br>R: AGGGTGTAAAACGCAGCTCA
Runx2-OT1	F: AAGCCATCCCACTCCTTCGGTAG<br>R: TCCAGCCCTGCCACATCAGAG
Apbb2-OT2	F: GAATCTCGCTGCCGCTGCTC<br>R: GCTCCCAGGTCTCGCAGTCC
Kcnab1-OT1	F: TCCTCCTCGGACTGCTGCTTG<br>R: CCTTCCCTGCCTCCCTGCTC
TVX1	F: GCCGAGCCTCAGCCACATAATC<br>R: TGTGTCTCTTACTCCTGCGTCTCC
Sema5a-OT1	F: AGCCGCACGCATTTCATACCC<br>R: ATCACTCCCCTCCAACAGTTCCTC
Dnajb3-OT5	F: GGCATCATCACCATCGGAACTCC<br>R: GACTCGGGCATTCATCACACACTC

Socs6-OT1	F: CGATGAAGCACAGTGAGTGAGACC<br>R: GACACCATGCAGACACCTGACAG
Rad51b-OT1	F: GAAACTGAAGGCGGCCATGG<br>R: GCTCTGCTCCGAAGTGAGGT
Usp45-OT1	F: ACCTGCTGAGTGATGGTGGC<br>R: AACCTFCTCTGTGGACCAGG
Rara-OT2	F: CCCCATGCCCCGAGGAAGAG<br>R: CCAAGCAGCCACAGTCAGAAGAG
Macrod2-OT2	F: AGGCTCTGAGGTGCTGCTGTG<br>R: CTCGGGAGGGAAAAGTGCAAAGG
LINC1742	F: GCATGTATGTCAGGGCACCAGATG<br>R: GCATGTATGTCAGGGCACCAGATG
IL-1β	CACTACAGGCTCCGAGATGAACAAC<br>TGTCGTTGCTTGGTTCTCCTTGTAC
IL-6	CTCCCAACAGACCTGTCTATAC<br>CCATTGCACAACTCTTTTCTCA
IL-18	AGACCTGGAATCAGACAACTTT<br>TCAGTCATATCCTCGAACACAG

Table S2. Quality assessment of lncRNA sequencing.

Sample name	Raw reads	Clean reads	Raw bases (G)	Clean bases (G)	Error rate (%)	Q20 (%)	Q30 (%)	GC content (%)
A 1	90261956	87689564	13.54	13.15	0.02	98.38	94.98	56.45
A 2	78390842	75957418	11.76	11.39	0.02	98.50	95.26	53.99
A 3	91410330	88895678	13.71	13.33	0.02	98.43	95.05	52.68
B 1	78957812	76018072	11.84	11.40	0.02	98.39	94.95	53.57
B 2	95474126	91590888	14.32	13.74	0.02	98.39	95.03	55.75
B 3	88416854	84915634	13.26	12.74	0.02	98.83	96.25	53.98
C 1	80497734	77584648	12.07	11.64	0.02	98.68	95.90	54.16

C 2	89036448	84586346	13.36	12.69	0.02	98.90	96.34	54.46
C 3	81207732	77293500	12.18	11.59	0.02	98.45	95.10	53.38
